# Training in new forms of human-AI interaction improves complex working memory and switching skills of language professionals

**DOI:** 10.3389/frai.2023.1253940

**Published:** 2023-11-17

**Authors:** Anna-Stiina Wallinheimo, Simon L. Evans, Elena Davitti

**Affiliations:** ^1^Centre for Translation Studies, Faculty of Arts and Social Sciences (FASS), University of Surrey, Guildford, United Kingdom; ^2^School of Psychology, Faculty of Health and Medical Sciences (FHMS), University of Surrey, Guildford, United Kingdom

**Keywords:** AI-related technologies, automatic speech recognition (ASR), interlingual respeaking (IRSP), human-AI interaction (HAII), cognition, executive function (EF), working memory (WM)

## Abstract

AI-related technologies used in the language industry, including automatic speech recognition (ASR) and machine translation (MT), are designed to improve human efficiency. However, humans are still in the loop for accuracy and quality, creating a working environment based on Human-AI Interaction (HAII). Very little is known about these newly-created working environments and their effects on cognition. The present study focused on a novel practice, interlingual respeaking (IRSP), where real-time subtitles in another language are created through the interaction between a human and ASR software. To this end, we set up an experiment that included a purpose-made training course on IRSP over 5 weeks, investigating its effects on cognition, and focusing on executive functioning (EF) and working memory (WM). We compared the cognitive performance of 51 language professionals before and after the course. Our variables were reading span (a complex WM measure), switching skills, and sustained attention. IRSP training course improved complex WM and switching skills but not sustained attention. However, the participants were slower after the training, indicating increased vigilance with the sustained attention tasks. Finally, complex WM was confirmed as the primary competence in IRSP. The reasons and implications of these findings will be discussed.

## 1 Introduction

In the language industry, which is currently one of the fastest growing industries (CSA Research, 2023), AI-related technologies, including automatic speech recognition (ASR) and machine translation (MT), have been created to automate repetitive and time-pressured tasks. However, these technologies are currently not sufficiently accurate to be used alone: human input is needed for sense checking and quality control. Humans are, therefore, responsible for monitoring and possibly correcting the written output produced by technology through Human-AI Interaction (HAII). Thus, AI-related technologies intended to extend and improve human efficiency are increasing the number of tasks people deal with, leading to new cognitive environments for professionals in the language industry and presenting new cognitive challenges and requirements.

In this paper, which draws on a wider experiment carried out within the framework of the ESRC-funded SMART project (*Shaping Multilingual Access through Respeaking Technology*, ES/T002530/1, 2020–2023), we will focus on a novel practice that relies on HAII, namely interlingual respeaking (IRSP). In IRSP, real-time subtitles in another language are created through the interaction of a human and ASR software (Davitti and Sandrelli, 2020; Pöchhacker and Remael, 2020). IRSP is a cognitively demanding real-time process where a language professional simultaneously translates incoming spoken language while adding punctuation and content labels orally, as well as applying any editing needed to ASR, which turns what they say into subtitles (Davitti and Sandrelli, 2020; Pöchhacker and Remael, 2020). This is a multi-step process where humans and technology need to work together to be able to produce highly-accurate subtitles in a timely manner.

Since the early 2000s, respeaking has been widely employed to produce intralingual subtitles (i.e., in the same language) for d/Deaf and Hard-of-Hearing audiences (Romero-Fresco, 2011). The recent worldwide increase in audiovisual content has led to an ever-increasing demand for making this content accessible across languages and in real time—hence the rise of interlingual respeaking (i.e., from one language to another), which adds language transfer to the traditional respeaking practice.

Pöchhacker and Remael (2020) conducted a detailed theoretical analysis of the IRSP process to guide future studies into the competences and skills required to perform it. In this newly-created process and competence model, the cognitive resources required for the IRSP process are placed in the technical-methodological competence area (Pöchhacker and Remael, 2020). However, the required cognitive functions are based on a competence-oriented task analysis of the IRSP process rather than on experimental investigations. Thus, the current study aims to bring more depth and empirical evidence to these initial findings. To this end, we set up an experiment that included a purpose-made training course on IRSP. We investigated what cognitive resources measured prior to the training predicted higher IRSP accuracy post-training. As part of the investigation, we also explored how the training course affected human cognition, executive functions (EF) and working memory (WM), in particular. We were interested in these cognitive functions as distributed cognition (DCog) posits that integrating technological tools with internal cognitive resources can increase the mental workspace available (Kirsh, 1995; Wallinheimo et al., 2019). However, little is known about how HAII affects human cognition, particularly when applied to real-time practices involving multiple tasks conducted under severe time constraints (as required by IRSP). As HAII becomes more common in the fast-evolving modern workplace, knowledge around the links to an individual's cognitive processes is needed to allow for people-centered and responsible AI.

### 1.1 New cognitive environment based on DCog

IRSP creates a new cognitive environment where human cognition is distributed to the outside world by relying on technological tools i.e., the use of ASR. DCog aims to understand the organization of human cognitive systems by extending what is traditionally considered cognitive beyond an individual doing the task to include interactions between the people involved in the process and the external resources e.g., technological tools in the environment (Hutchins, 1995; Hollan et al., 2000). In DCog, a new broader unit of cognitive analysis is created that includes a network of technologies and actors leading to a process that coordinates internal processes in the minds of humans working together, with external representations relying on external artifacts. According to Vallée-Tourangeau and Vallée-Tourangeau (2017), thinking can be seen as a cognitive process that develops in time and space and leads to a new cognitive event, for example, a solution to a problem. These cognitive events emerge from cognitive interactivity, which the authors define as “the meshed network of reciprocal causations between an agent's mental processing and transformative actions she applies to her immediate environment to achieve a cognitive result” (Vallée-Tourangeau and Vallée-Tourangeau, 2017).

Thus, novel forms of HAII give rise to new working environments that impact existing cognitive processes in distinct ways. Several experimental studies have explored individual problem solving to examine cognitive interactivity, yielding valuable insights into the use and benefits of distributing human cognition, with a particular focus on the cognitive needs of the individual. Wallinheimo et al. (2019) found that when evaluative pressure is experienced to complete a cognitive task, there are additional demands on the existing limited WM resources. However, some of these WM limitations, caused by the additional worry of performing well, can be compensated by offloading the cognitive process to the external environment by using pen and paper or other external artifacts (Wallinheimo et al., 2019). This is in line with Risko and Gilbert (2016), who argue that cognitive offloading reduces the overall cognitive demand on the individual (Risko and Gilbert, 2016). Additionally, Kirsh (2010) claimed that there are cost-benefit considerations for cognitive interactivity, and as a result, cognitive processes go to wherever it is easier to perform them. It might be easier to understand a complicated sentence by drawing a picture of it, to visualize it externally rather than just thinking internally in the head alone. Thus, the overall cognitive cost of sense making to understand the sentence is reduced with the help of drawing a picture (Kirsh, 2010). Finally, cognitive interactivity can extend WM resources when there is a cognitive need to do so (e.g., less efficient WM capabilities) (Webb and Vallée-Tourangeau, 2009).

When it comes to IRSP, however, both the human and the machine have equally important roles to make the IRSP process work as both are required to work in synchrony for the creation of accurate interlingual subtitles. This is a form of human-autonomy teamwork (HAT) where humans work interdependently with an autonomous agent (i.e., ASR) focusing on a set of tasks toward the shared goal of producing interlingual live subtitles (O'Neill et al., 2022). Thus, during the IRSP process, human cognition is distributed with the use of ASR not due to a cognitive need of the language professional (i.e., reduced WM capacity, cost-benefit considerations, or cognitive offloading), but rather as a requirement inherent to the IRSP process itself. This leads to a distinct cognitive environment that sets it apart from the experimental studies mentioned earlier.

### 1.2 IRSP and simultaneous interpreting

IRSP is a new practice, and empirical investigations into the cognitive processes involved are in their infancy. As a real-time language-related practice, IRSP shares many aspects with simultaneous interpreting (SI), which is widely acknowledged as one of the most cognitively challenging tasks of human cognition (Babcock and Vallesi, 2017). Hence, we have drawn upon the existing SI literature as the starting point of our investigation, recognizing its relevance in understanding the cognitive intricacies of IRSP. When simultaneously interpreting, an interpreter needs to concurrently comprehend auditory material in one language while producing the same content in another language. Executive functions (EF) are heavily involved in this process. EFs are a set of cognitive processes that are needed for the cognitive control of human behavior. The three most postulated areas of EF are: shifting between tasks or mental sets (shifting), information updating and monitoring in WM (updating), and inhibition of prepotent responses (inhibition) (Miyake et al., 2000). SI requires both short-term memory and WM resources to keep the required information active and to be able to manipulate it throughout the SI process (Timarova, 2007; Aben et al., 2012; Mellinger and Hanson, 2019). Additionally, for the simultaneous interpreter to keep control of incoming information and avoid mixing languages, effective recall and attentional and cognitive control are needed (Christoffels and de Groot, 2005). It is evident that the parallel processing of input and output information taxes different neurocognitive resources.

During SI, there is maximal use of linguistic and cognitive control hubs compared to simultaneous repetition (Hervais-Adelman et al., 2015). It does not, therefore, come as a surprise that professional interpreters have shown clear advantages in terms of improved memory and EF functions. Professional interpreters seem to exhibit greater WM capacity when compared with comparison groups (i.e., students and non-interpreters) (Mellinger and Hanson, 2019). In a study exploring what professional background can best support respeaking, Szarkowska et al. (2018) suggested that interpreters achieved higher accuracy rating scores in IRSP when compared with translators, and people with no interpreting or translation experience. The difference in IRSP performance was moderated by WM capacity (Szarkowska et al., 2018). In addition, Morales et al. (2015) found that professional SI participants were better at maintaining, updating, and processing of information in the WM when measured with a N-back Task, compared to individuals who were fluent in the second language but had no professional experience. Finally, studies into bilingualism have found that EF skills, including mental flexibility, task switching, and attentional and inhibitory control, are enhanced compared to monolinguals (Soveri et al., 2011; Strobach et al., 2015).

### 1.3 The effects of interpreter training on WM and performance

Previous studies have shown that interpreter training can boost participants' WM performance (Macnamara and Conway, 2016; Babcock et al., 2017; Chmiel, 2018). Also, nine months of SI training have been shown to cause structural and functional brain changes in temporoparietal, frontostriatal, and temporoparietal brain circuits (Van de Putte et al., 2018). Chmiel (2018) conducted a longitudinal study over 2 years where WM performance (measured with a reading span task) of professional interpreters, interpreter trainees, and bilingual controls, was investigated. The professional interpreters outperformed on WM tasks at baseline. However, after a 2-year interpreter training, the trainees scored higher on WM tasks (Chmiel, 2018). In another longitudinal study, the WM performance (measured with backward span, reading span, and operation span) of 50 American Sign Language (ASL) simultaneous interpreting students was measured before and then 2 years after a sign language interpreting course: the students' simple WM (i.e., backward span) was enhanced, but not their complex WM (i.e., reading span and operation span). Thus, SI training appears not to improve memory skills that require concurrent storing and processing of information (Macnamara and Conway, 2016). Likewise, Babcock et al. (2017) conducted a longitudinal investigation and found that 2 years of SI training was associated with positive cognitive changes in verbal short-term memory, measured with a letter span task. There were no significant findings in relation to operation span and symmetry span (complex measures of WM).

In the SMART project's experiment, a customized training course was created to ensure that all participants, i.e., language professionals from various backgrounds, received equal exposure to IRSP before undergoing testing (see Materials—The IRSP Upskilling Training-For-Testing Course). Given the hybrid nature of IRSP, sharing many similarities with SI, we decided to investigate cognitive enhancement between the start and end of the course. Due to the multi-step nature of IRSP, simple WM was not part of these analyses.

### 1.4 Positive cognitive changes through specific skills training

Several studies in domains other than Translation and Interpreting Studies have investigated how training in specific skills can lead to positive cognitive changes. One of these critical research areas is online gaming and action games, in particular, where cognitive functions can be enhanced by the extensive practice of playing the games (Boot et al., 2011). Online action games are comparable to IRSP in that both involve multiple simultaneous actions that occur in real time. EF skills in these domains involve processing complex situations involving simultaneous and sequential tasks with quick, real-time switches between them (Logan and Gordon, 2001). Frequent video gaming has been found to benefit the development of EF skills, particularly attention skills, task switching, and WM (Alho et al., 2022) suggesting similarities between the cognitive requirements of online gaming and IRSP. Many studies have shown that when young adult non-gamers are trained in action video games, their visual attention skills, task switching, and multiple object tracking improve (Green and Bavelier, 2003; Strobach et al., 2012; Oei and Patterson, 2013). In another study, Parong et al. (2017) tested a custom-made online game (Alien Game) that focused on EF skills, concluding that playing 2 h of the online game when compared to a control game could improve shifting skills (Parong et al., 2017).

### 1.5 Study hypotheses

The current study is looking to investigate how cognitive processes of language professionals are affected when working on cognitively demanding real-time multi-step processes that rely on HAII. To this end, we investigated how cognitive resources measured at baseline i.e., reading and digit span (WM), N-back (a measure of maintaining, updating, and processing of information in the WM), switching skills, and sustained attention were associated with high IRSP performance that was measured at the end of the IRSP training course. This was to further extend and substantiate the initial findings by Pöchhacker and Remael (2020), providing empirical evidence to some of the essential skills and competences required in the IRSP process. Additionally, we wanted to further explore how the purpose-built IRSP training course might affect the wider cognitive skills (complex WM, switching skills, and sustained attention) of language professionals. Notably, previous empirical findings on simultaneous interpreters have indicated that complex WM can be enhanced (Chmiel, 2018). Given the multi-step nature of IRSP, we anticipated similar advantageous effects and benefits in this domain as well.

Therefore we hypothesized that there would be a positive relationship between baseline complex WM resources and post-training IRSP accuracy (Hypothesis 1) in line with previous findings on SI (Timarova, 2007; Aben et al., 2012). With respect to N-back (WM) we predicted that it would be positively associated with high IRSP accuracy (Hypothesis 2). Previous findings have suggested that participants with professional SI experience outperform control participants with fluency in the second language but no professional experience of SI, on N-back performance (Morales et al., 2015). Also, given that several cognitive abilities in relation to IRSP have not been tested previously, this study took an exploratory approach to investigate these further. Hence, we investigated how simple WM, switching skills, and sustained attention might predict IRSP accuracy.

Furthermore, the effects of the training course on cognitive performance were examined. It was predicted that after attending a 5-week training course on IRSP, there would be an enhancement on complex WM (Hypothesis 3) as suggested by Chmiel (2018). We also hypothesized (Hypothesis 4) that switching skills would be improved after the training because of evidence from other cognitively similar domains, online gaming in particular (Parong et al., 2017; Alho et al., 2022). Our final hypothesis (Hypothesis 5) was that sustained attention would improve between the start and end of the training course as many studies in bilingualism have highlighted that attentional and inhibitory control can be improved when compared to monolinguals (Soveri et al., 2011; Strobach et al., 2015).

## 2 Materials and methods

### 2.1 Participants

Fifty-one language professionals with English, French, Italian, or Spanish as their mother tongue participated in this study (*M*age = 40.12 years, *SD* = 10.97 years). There were eight males (*M*age = 37.38 years, *SD* = 10.93 years) and 43 females in the study (*M*age = 40.63 years, *SD* = 11.51 years). The participants had a minimum of 2,000 h of professional experience in one or more language-related practices: spoken language interpreting (consecutive) 58.82%; spoken language interpreting (simultaneous) 52.94%; written translation 94.12%; pre-recorded subtitling 58.82%, and/or live subtitling 21.57%. The participants were grouped based on their language directionality: French (nine working into English and eight working into French); Italian (16 working into Italian and one working into English); and Spanish (eight working into Spanish and nine working into English).

### 2.2 Materials

#### 2.2.1 The IRSP upskilling training-for-testing course (“Advanced introduction to interlingual respeaking”)

This paper focuses on data collected before and after participants completed a bespoke 25-h upskilling course, delivered online over 5 weeks and in a self-taught manner. The course had the dual purpose of collecting data for the study (hence training-for-testing) and placing all participants on a level playing field in relation to this practice by providing them an “Advanced introduction to IRSP.” Due to the innovative nature of the practice and the limited number of fully trained professionals available, the study team designed the course to cater to language professionals from diverse walks of life, each bringing unique skills to this emerging field. To this end, the course broke down interlingual respeaking into three key modules: on technology, particularly exploring the main components of speech recognition software (Dragon Naturally Speaking v 15) and its functioning; intralingual practice, i.e., in the same language; and interlingual practice, i.e., into another language. The course proceeded through four sequential blocks that guided the learners through the steps required for IRSP gradually: (1) Simultaneous listening and speaking/translating and software-adapted delivery (i.e., how to adjust one's voice and prosody to ASR for optimal recognition); (2) Adding punctuation and related strategies for chunking and dealing with speed; (3) Software optimization and preparation prior to a respeaking task for accuracy; (4) Error correction via different methods. Learning proceeded through alternation of theory and practical exercises, designed to train each procedural skill firstly independently then in combination with others, in an incremental way. Participants performed each task first intralingually, then interlingually, before moving on to the next one, which allowed the participants to train in an incremental manner across a predetermined sequential order. Each task had to be completed before participants were permitted to proceed to the next one. At the end of the course, participants were tested on both intralingual and interlingual respeaking.

### 2.3 Cognitive measures

#### 2.3.1 WM (reading span task)

The reading span task (RST) is a complex memory span task including a processing component (lexical decision: judging the correctness of sentences) and a storage component (memorizing a series of words for subsequent recall) (Daneman and Carpenter, 1980). RST is widely used and adapted for verbal WM and cognitive processing investigations. It focuses on the active updating and monitoring of information in WM. Before the actual RST comprising 12 blocks, there were three practice trials. The task contained between 2 and 5 sentences in each block, and the participants were asked to judge the correctness of the sentences (e.g., “The surgery's giraffe is arriving after 20 min to open the doors” or “The mother rushed to the school to pick up her daughter”). In the storage component, there were between 2–5 words (e.g., “pet” and “bug”) to be recalled later. The primary output measure of the RST was the recall proportion of the words remembered (i.e., storage component of the task). The score on the correctness of sentences was not measured. It was used to make sure that the participants were paying attention to the task. The same RST was used during the pretesting and post-testing phases of the experiment. However, the sentences and words used were different during the pretesting and post-testing stages to avoid any practice effects. The participants completed an online version of the RST that was created in Pavlovia.

#### 2.3.2 WM (digit span task)

The digit span task (DST) is a simple memory span task. Unlike the complex WM measure that measures both processing and storage of WM, simple memory span task focuses on WM storage only. In this task, a person is presented with a sequence of digits (starting three digits) and asked to repeat the sequence. Participants do three conditions as part of the DST: forward span where the digits are recalled in the same order, backward span where the participants need to recall the digits in the backward order, and then recalling of digits in an ascending order involving the participant to sequence the numbers from the lowest to the highest. The number of digits increases 1 at a time (two trials for each span) until the participant fails on both trials. The longest remembered sequence is the person's digit span for that condition. This task was also created in Pavlovia and the participants completed it online.

#### 2.3.3 Switching skills

Switching skills were measured with a plus-minus task which measures switching between simple mathematical operands of addition and subtraction (Miyake et al., 2000). This function focuses on shifting back and forth between multiple tasks or mental sets and it can also be called attention switching and task switching. The participants started with addition, moving into subtraction, and finished with a task where they alternated between additions and subtractions. All the numbers used in the task were two-digit numbers (from 10 to 99), and they were only used once. The numbers (30 per condition, presented in a vertical column) were randomly mixed to form the three conditions (i.e., addition, subtraction, and switch: alternation between addition and subtraction). Participants worked their way down the column and entered the answer in the space next to it, in Qualtrics. Time taken was measured, when they completed a column, Qualtrics moved on to the next condition. First, they added the number three to each number (e.g., 83 + 3, addition condition. Then, for the second condition, they subtracted the number three (e.g., 75 – 3). Then, they alternated between addition and subtraction of a 3 as they worked their way down the column, in the third condition. A switching cost was calculated where the non-switch completion time (an average of time taken to complete the addition and subtraction conditions) was subtracted from the time to complete the switch condition). The same plus-minus task was used during the pretesting and post-testing. However, the randomization of the double-digit numbers was different during the pretesting and post-testing stages of the experiment to mitigate any practice effects.

#### 2.3.4 Sustained attention to response task

In this computer-based go/no go task, participants are required to make a response every time they see a number (1–9) by pressing a key, except when that number is three, in which case they must withhold their response (Robertson et al., 1997; Manly and Robertson, 2005). During the sustained attention to response task (SART) task, inhibitory control is necessary to discriminate between relevant and irrelevant distractors (Manly and Robertson, 2005). Sustained attention is required for constant monitoring of the task. Five blocks of 45 trials each (225 trials in total) were presented visually over 4.3 min. The participants responded with a key press to each digit except when the number three appeared on the screen (25 times) when they had to withhold their response. Number three was distributed throughout the 225 trials in a quasi-random way. The participants used their preferred hand to respond and were told to focus on accuracy and speed equally. Before starting the actual task, each participant did a practice that comprised eighteen numbers, two of which were the target number three. The primary measure of the SART task was the proportion of targets (“3”) to which the participants successfully withheld their response. We also measured the average reaction time (in seconds) of the participants. This online version of the task was created in Pavlovia.

#### 2.3.5 N-back task

N-back is a widely used measure for assessing WM, which requires the participant to maintain, continuously update, and process information (Kirchner, 1958). N-back is commonly used to measure WM monitoring and updating, while minimizing the storage component (Morales et al., 2015). Hence, it is used to evaluate the updating function of the Miyake's model of EF (Miyake et al., 2000) and is linked to the central executive (CE) of the Baddeley's model of WM where it refers to the monitoring of incoming information for task relevance. The information that is not needed for the completion of the task is updated with the new information as part of the CE (Baddeley and Hitch, 1974; Baddeley, 2012). In the current study, the participants completed two practice blocks before the actual task: one for the 0-back and the second one for the 2-back. Participants were instructed to monitor a series of stimuli and to respond whenever a stimulus was presented that was the same as the one presented *n* trials before. The letters that acted as the stimuli were presented for 500 ms followed by a 2,500 ms black period. The N-back Task had an equal number of blocks for 0-backs (10 blocks) and 2-backs (10 blocks). Participants either matched a letter to the target (0-back) or indicated whether it matched with one presented 2 before (2-back) by pressing a key on the keyboard. Average accuracy was calculated for 0-back blocks and 2-back blocks. This was an online task created in Pavlovia.

### 2.4 Procedure

**Figure 1:**
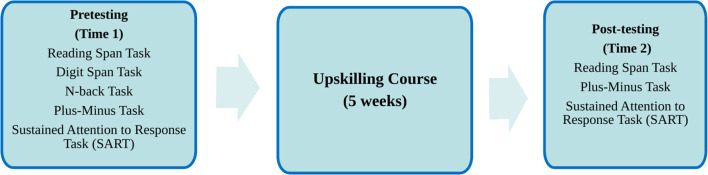
The procedure of the experiment (cognitive component).

Due to the pandemic, this study was entirely conducted online and advertised on the SMART project homepage and dedicated social media account. Any interested language professionals were sent an eligibility questionnaire that focused on language eligibility (i.e., English paired with Italian, Spanish, and/or French, with at least one of these languages as their mother tongue), professional eligibility (i.e., a minimum of 2,000 h of professional experience in language-related practices, namely consecutive and/or dialogue interpreting, translation, live and/or offline subtitling), and correct equipment specifications (laptops, headset, and microphone). Participants who met all the eligibility criteria were sent a link to the study, comprising cognitive tasks created in Pavlovia and Qualtrics. Consent was given by the participants before starting. The experiment started as soon as the participant opened the link, and it took the participant through the whole pretesting phase of the experiment in one go (Figure 1 summarizes the procedure of the experiment in relation to the cognitive component analyzed in this paper). Before beginning the data collection process, we integrated Pavlovia and Qualtrics and tested it carefully to ensure that all participants would go through the same experimental steps. Despite the lack of strict experimental conditions, we aimed for a rigorous approach. We also had a pre-testing call with each participant to explain the cognitive testing steps and answered any questions. Participants were pretested on several cognitive abilities, specifically WM (including reading span, digit span, and N-back), switching skills, and sustained attention, with a duration of 40 min. The pretesting was followed by the 25-h upskilling course. The participants were provided with a link to the upskilling course, which was hosted on Moodle, and worked on the four different blocks independently online. Subsequently, they were tested on their intra and interlingual respeaking performance. In the current study, only the interlingual respeaking performance was used as a basis of accuracy for our investigations. The accuracy of the subtitles thus produced was assessed using the NTR model (Romero-Fresco and Pöchhacker, 2017—see Analytic plan below), which focuses on the type of errors made while performing IRSP. After the training-for-testing, participants were sent a follow-up link to complete three post-testing cognitive measures (reading span, switching skills, and sustained attention), which took ~25 min and were delivered via the same platform as the one used for pre-testing measures (Pavlovia). Upon completion of the cognitive tasks, participants were asked to take part in a final evaluation questionnaire to gather information and feedback about the course, after which they were debriefed and thanked for their participation.

### 2.5 Analytic plan

Our study involved a two-part statistical analysis that enabled us to examine our five hypotheses. In the first phase of the analysis, we concentrated on IRSP accuracy measured at the end of the training and how it was predicted by baseline cognitive abilities.

To assess the accuracy of IRSP performance, we employed the NTR Model (Romero-Fresco and Pöchhacker, 2017) which specifically focuses on the nature of errors committed by language professionals while producing interlingual live subtitles via respeaking. The NTR formula distinguishes between software-related recognition and human translation errors, including content-related errors (i.e., omissions, additions, and substitutions) and form-related errors (grammatical correctness and style). NTR accuracy is based on the following formula: NTR = [(*N*-*T*-*R*)/*N*] × 100%, where *N*, number of words; *T*, translation errors; and *R*, recognition errors. Errors get different scores depending on their severity. Minor errors are penalized with a −0.25-point deduction as they do not impair comprehension. Major errors, however, can cause confusion and information loss, and are penalized with a −0.50-point deduction. Finally, critical errors can introduce false or misleading information, and therefore they are penalized with a −1.0-point deduction. Intralingual subtitles (i.e., in the same language) are required to reach an accuracy rate of 98%. A similar accuracy rate is suggested for interlingual subtitles (i.e., in a different language), although this benchmark has not been validated yet.

Multiple regression was used to investigate what predicted post-training IRSP performance. Our multiple regression model predictors were reading span (WM), digit span (WM), N-back (WM), switching skills, and sustained attention, at baseline.

In the second (longitudinal) part of the analysis, we looked at the effects of the IRSP course on three cognitive abilities that were measured both before and after the course (reading span, switching skills, and sustained attention) using a repeated-measures within-subjects design by looking at changes in cognitive performance between these two time points.

## 3 Results

Before conducting the actual statistical analyses, we investigated whether the data was normally distributed. Shapiro-Wilk's test was non-significant for all the variables, suggesting that all the data were normally distributed. We also viewed histograms to confirm normality and checked box plots. No extreme values or outliers were found. The test of sphericity was non-significant (*p* > 0.05) indicating that the assumption of sphericity was met. Table 1 summarizes all the descriptive statistics (pretesting and post-testing data). Based on the NTR Model, the participants' average IRSP accuracy was *M* = 95.37% and *SD* = 1.5%, indicating that the accuracy was lower than the recommended 98% for intralingual live subtitles (i.e., in the same language). Table 2 includes all the data for the multiple regression analysis.

**Table 1:** Descriptive statistics for all the variables (pretesting and post-testing data).

**Measures**	**Pretesting**			**Post-testing**		
	***M***	***SE***	***P*** **-value**	***M***	***SE*** **(SD)**	***P*** **-value**
RST	0.83	0.02	0.05	0.88	0.02	0.05
Plus-minus (s)	22.90	2.95	0.02	14.55	1.85	0.02
SART (accuracy)	0.96	0.003	0.50	0.96	0.004	0.50
SART (RT in s)	0.37	0.008	0.06	0.39	0.009	0.06
NTR accuracy (%)				95.37	(1.5)	

RST, reading span task (WM); Plus-minus, plus-minus task (switching skills measured with a switching cost in seconds); SART, sustained attention to response task (sustained attention). NTR accuracy was only measured after the training course.

**Table 2:** Multiple regression model with IRSP accuracy as the criterion variable.

**Measures**	**Unstandardized**	**Standardized**	***t***	***P*-value**
	**Coefficients (B)**	**Coefficients (beta)**		
RST	0.03	0.32	2.33	0.03
DST (1)	0.00	−0.04	−0.25	0.80
DST (2)	0.00	0.23	1.43	0.16
DST (3)	−0.00	−0.11	−0.74	0.47
N-back	0.02	0.20	1.46	0.15
Plus-Minus (s)	0.00	−0.14	−1.04	0.30
SART (accuracy)	−0.14	−0.21	−1.54	0.13

RST, reading span task (WM); DST, digit span task (WM); DST 1, forward span; DST 2, backward span; DST 3, ascending numerical order; N-back, N-back task (WM); Plus-minus, plus-minus task (switching skills measured with a switching cost in seconds); SART, sustained attention to response task (sustained attention).

### 3.1 Cognitive predictors of IRSP accuracy

A multiple linear regression was conducted to predict NTR accuracy based on pre-testing (baseline) reading span, digit span, N-back, switching skills, and sustained attention. The multiple regression model was significant *F*_(7, 42)_ = 2.27, *p* = 0.04 and the adjusted *R*^2^ indicated that 15.4% of the variance in the IRSP accuracy was explained by the model. There was a significant positive relationship (β = 0.32) between the participants' reading span (a complex WM measure) and their IRSP accuracy. However, the other predictors (i.e., digit span, N-Back, switching skills, and sustained attention) were not statistically significant, as *p* > 0.05 (see Table 2).

### 3.2 Pretesting and post-testing data

To investigate the possible effects of the IRSP training course on cognitive performance, we compared the cognitive performance of participants from before (pretesting—baseline, T1) to after the course (post-testing, T2). Our variables were reading span (a complex WM measure), switching skills, and sustained attention.

#### 3.2.1 Complex WM (reading span)

**Figure 2:**
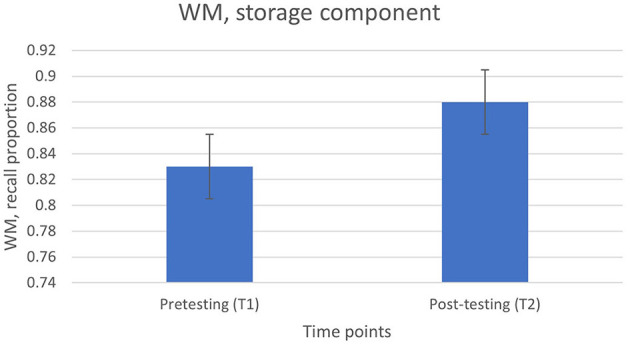
WM at time points 1 and 2.

Complex WM from the RST task improved from T1 to T2, *F*_(1, 46)_ = 4.0, *p* = 0.05 (from *M* = 0.83, *SE* = 0.02 to *M* = 0.88, *SE* = 0.02), suggesting that the IRSP training course might have improved the WM resources of the language professional (Figure 2).

#### 3.2.2 Switching skills

**Figure 3:**
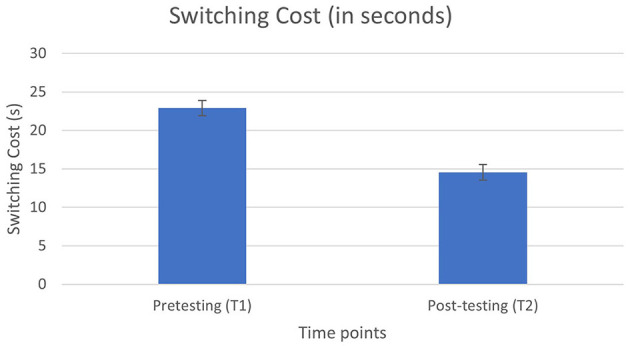
Switching cost at time point 1 and at time point 2.

Switching skills (switching cost in seconds from the plus-minus task) improved from T1 to T2, *F*_(1, 49)_ = 6.42, *p* = 0.02 (from *M* = 22.90 s, *SE* = 2.95 s to *M* = 14.55 s, *SE* = 1.85 s) showing that the IRSP training course might have enhanced the participants' switching skills (Figure 3).

#### 3.2.3 Sustained attention

There was no significant change in sustained attention (SART), accuracy between T1 and T2, *p* > 0.05. However, there was a marginally significant difference in SART reaction times (seconds) between T1 (*M* = 0.37, *SE* = 0.008) and T2 (*M* = 0.39, *SE* = 0.009), *F*_(1, 48)_ = 3.60, *p* = 0.06, with the means indicating that the participants were slower on the SART task post-training.

## 4 Discussion

AI-related technologies are developed with the goal of augmenting and improving human efficiency. However, at present, human involvement is still necessary to oversee and modify the output generated by these technologies. As a result, this places an additional burden on humans, increasing the number of tasks they are responsible for managing. When working with AI-related technologies such as ASR, a new working environment is created in the form of HAII where human cognition is partly distributed to the outside world. IRSP is a case in point, where very little is known about its process and the human cognitive requirements for this newly-created HAII, and in turn how cognitive processes are affected by engaging with it. To this end, we focused on IRSP, a cognitively demanding process, to study the links between this novel form of HAII and human cognition. We investigated what baseline cognitive abilities predicted higher IRSP performance after a 25-h upskilling course. We also explored whether the course would improve the EF and complex WM of language professionals. We focused on these cognitive areas, as previous work on SI and bilingualism had suggested their involvement and highlighted the possibility of improvements within these domains.

Our hypotheses were partly supported. There was a positive relationship between complex WM resources (reading span) measured at baseline and subsequent post-training IRSP performance, confirming our first hypothesis. Complex WM was the only significant finding in relation to the five cognitive predictors of high IRSP performance under investigation (complex WM, simple WM, N-back, switching skills, and sustained attention), clearly emphasizing complex WM as a leading competence required for accurate IRSP performance. These results are in line with existing findings suggesting that WM resources are required to manage the cognitively demanding process of SI, a practice that shares many similarities with IRSP (Timarova, 2007; Aben et al., 2012; Mellinger and Hanson, 2019). Furthermore, this finding complements the process and competence model by Pöchhacker and Remael (2020) by bringing empirical evidence regarding complex WM resources and their role in achieving high accuracy in IRSP. On the other hand, simple working memory, measured with a digit span task (focusing on WM storage only), was not a predictor, suggesting that to perform well in IRSP, simultaneous processing and storage of WM are both required rather than just WM capacity *per se*. The multi-step and real-time nature of IRSP is likely the reason why both processing and storage are required, to enable professionals to keep up with the task and reach high accuracy.

There were no significant findings in relation to the third WM measure (N-back Task) failing to support our second hypothesis. These findings are in contrast with Morales et al. (2015) who suggested that participants with SI experience performed better in monitoring and updating, measured with the N-back Task, when compared to a control group. It is possible that the use of ASR as part of the IRSP process alters the WM requirements, leading to a stronger need for simultaneous processing and storing of information (measured with a complex WM task) rather than just updating of information. The monitoring and possible editing of the ASR output might change the focus of the language professional and therefore create an environment where strong complex WM resources are more important. Also, it should be noted that both the N-back and digit span tasks use numeric rather than word stimuli. This could have contributed to the lack of effects and future studies should consider adapting the tasks to explore whether this is important in this context.

Additionally, our findings highlighted that switching skills were not positively associated with better post-training IRSP performance. This finding is somewhat surprising as past findings have indicated that bilinguals have enhanced switching skills compared to monolinguals (Soveri et al., 2011) and that SI improves the ability to coordinate multiple tasks in dual-task situations (Strobach et al., 2015). It might be that the plus-minus task used here (as a pure “switching” measure) does not capture the complexity and variety of the multitasking skills required by IRSP, and a longer and more advanced task requiring switching between multiple sources of information would have found effects. However, time constraints precluded use of such task here.

Similarly, sustained attention was not associated with high IRSP accuracy either. IRSP is a time-pressured process with high levels of task demand. Its continuous demands on cognitive resources meant that we expected sustained attention to be a predictor, but this was not supported. However, continuous performance tasks such as SART require subjects to maintain attention during a monotonous, repetitive, task with low levels of demand. Again, this does not reflect the IRSP environment. It seems that the ability to avoid distraction and mind-wandering during such a task is not a predictor of IRSP performance, but this is perhaps not surprising when IRSP imposes such high cognitive demands. Also, it should be noted (as discussed further below) that SART performance was very high across the sample, so ceiling effects could explain the lack of predictive utility. Continuous attention when task demands are high might be a more appropriate measure which should be tested in future work. In sum, results highlight the importance of complex WM as a predictor of IRSP accuracy, with simple WM, switching skills, N-back (maintaining, updating, and processing of information in WM), and sustained attention not being significant predictors. Future studies should explore the role of complex WM in more detail and include alternate measures of the other cognitive skills to confirm the current findings.

When looking at the effects of the IRSP training course on cognitive performance, our results suggested that complex WM improved between the start and end of the training course, indicating that there can be possible cognitive enhancements with IRSP training, confirming our third hypothesis. These findings also highlight the malleability of WM resources with the help of a training course, confirming existing findings around effects of cognitive training (Morrison and Chein, 2011; Pappa et al., 2020). As aforementioned, the use of ASR might change the cognitive environment with more emphasis on the requirement of complex WM resources. By attending the training course, complex WM resources were challenged and seem to have improved. These findings are in line with Chmiel (2018) who confirmed that after a 2-year training in interpreting, the interpreter trainees scored higher than professional interpreters on complex WM tasks. However, these results do not align with previous investigations by Macnamara and Conway (2016) who reported that a 2-year SI training in ASL did not improve complex WM of ASL simultaneous interpreting students, but the training did enhance simple WM resources. Similarly, there were no significant findings in relation to complex WM measures but to simple WM measures after 2 years of SI training in the Babcock et al. (2017) study where the performance of SI students was compared to translation students and non-language students as the control groups. It is possible that the multi-step nature of IRSP, including the use of ASR might explain the improvement of complex WM in our study in contrast to Babcock et al. (2017) and Macnamara and Conway (2016) studies. Any future studies should focus on looking at the complex WM resources of different groups of language professionals.

In the present study, although switching did not emerge as a predictor of post-training IRSP performance, switching skills were enhanced after the IRSP training course, confirming our fourth hypothesis. IRSP requires the language professional to actively switch between tasks, involving simultaneous interpreting, and monitoring of the ASR output. The design adopted in the IRSP training course has, therefore, facilitated the development of these skills among language professionals, possibly leading to their enhancement. Similar to complex WM, our findings support the idea that switching skills are malleable (Zhao et al., 2020) and can be enhanced with training. However, the shortcomings of the plus-minus task and the fact that it did not predict performance, means this result should be viewed with caution: it could be an artifact of task-specific practice effects. Nevertheless, these findings align with current research findings in online gaming as multi-step process activities (Parong et al., 2020; Alho et al., 2022). Frequent online gaming was found to benefit task switching (Alho et al., 2022) and shifting between competing tasks (Parong et al., 2017). Finally, our findings have clearly highlighted that new forms of HAII might increase the number of tasks the human needs to focus on; however, these findings also indicate potential cognitive benefits for the individual engaging in this complex practice.

Regarding our final hypothesis, which posited improvements in sustained attention accuracy, there were no significant findings, thus failing to support the hypothesis. The baseline SART accuracy was high (96.1%), possibly indicating that the SART task was easy for the language professional to complete because of possible previous experience in activities requiring sustained attention. Perhaps, there were ceiling effects and therefore, the accuracy could not be enhanced any further. However, when looking at the SART reaction times, the language professionals became slower (at trend) post-training. IRSP process fosters a behavior where the accuracy of the subtitles produced is imperative. This has perhaps led the language professional to be more prudent with their strategies while completing the SART task, leading to slower reaction times (RT). Similar pattern was found by Vallesi et al. (2021) who suggested that SART accuracy was improved with additional vigilance with the task. It is also possible that another type of attention is required in IRSP and that is why future research should focus on other types of attentional skills (e.g., divided attention).

In the present study, DCog is seen as the foundation of HAII. Clearly, a new cognitive environment is created with IRSP where parts of the human cognition is distributed with the help of ASR, leading to interactions between humans and technological tools. According to DCog, the use of external artifacts and technology have the potential to increase the workspace available for the human (Kirsh, 2010; Vallée-Tourangeau, 2013; Wallinheimo et al., 2019). However, it is not clear what happens during the IRSP process when technology does not work the way the language professional wants it to. During the IRSP process, the language professional might need to correct what has been produced by the ASR and it is possible that the human loses the sense of personal control over the situation (Ehrensberger-Dow and O'Brien, 2015). There can be additional worry and anxiety, leading to additional taxation of WM and hampered IRSP performance. Any future experimental IRSP studies should focus on these important aspects that allow humans and technology to work successfully together.

### 4.1 Limitations and future studies

Whilst we have revealed some interesting findings that advance literature, there are clear limitations. In IRSP, there are additional steps for the language professional at the core of the activity to monitor and ensure the accuracy of the subtitles produced in conjunction with ASR technology. We suspect that this might lead to an increased workload and cognitive load. However, we have not measured cognitive load as part of the present investigation. Future studies should focus on understanding how the different tasks requiring varying cognitive resources affect the human's cognitive load and whether this impairs respeaking performance and other cognitive performance. This approach could then be transferred to other real-time HAII practices witnessing high burnout risks (e.g., the financial sector and aviation industry), allowing for optimal performance without ignoring the needs of the individual involved.

From a methodological perspective, it is noteworthy that the entire study was carried out online due to the pandemic. Despite our efforts to create a seamless and well-integrated experience for participants, as detailed in the procedure section, variations in participants' individual testing environments during the experiment are possible. However, we took measures to minimize potential repercussions on the conduct of the experiment. We ensured that all tasks were organized within a clear and structured flow, complete with instructions. Moreover, we communicated directly with participants before the tests (via individual pre-testing calls), emphasizing the importance of completing them in a quiet environment without disruptions to avoid breaking the flow and getting distracted. We closely monitored the process by focusing on the reaction times and found no indications of participants not adhering to the provided instructions.

In addition, it is possible that there were practice effects on the cognitive tasks between the pre-testing and post-testing phases when reading span, switching skills, and sustained attention were measured. Our investigation is focused on language professionals who completed an upskilling course on IRSP. However, we have not compared our findings to a control group. Future research should focus on investigating any possible cognitive changes in relation to other similar types of training courses compared to the IRSP training of language professionals. Our study involved a professional sample with an older average age. Therefore, comparing our findings with other studies that mainly focus on student samples might be challenging. However, it is true to say that an older sample might be more motivated to participate in a study like this (Ryan and Campbell, 2021). Additionally, while we used cognitive measures that have been previously used in SI research, it is important to note that SI rely on a different degree of interaction with technology, and thus creates a different cognitive environment when compared to IRSP. As such, different cognitive measurements might be needed to effectively evaluate human-AI collaboration in this practice.

## 5 Conclusion

The present study has allowed us to complement and provide empirical evidence to the process and competence models by Pöchhacker and Remael (2020) by suggesting that complex WM resources are required to achieve high IRSP accuracy. These findings could be transferred to other similar real-time work processes involving humans and technology to highlight the importance of complex WM resources in comparable practices. Furthermore, our study adds to the growing literature on possible cognitive enhancements after a training course. We found that both complex WM and switching skills were improved with IRSP training, highlighting the fact that these skills can be trained and their possible malleability. The newly-created HAII environment of IRSP seems to lead to positive cognitive enhancements for the language professional. Whilst there might be an increased workload by monitoring and editing the output of ASR during IRSP, there seem to be clear cognitive benefits in doing so. However, more investigations are required to further understand the possible risk of burnout when working in real-time HAII practices to allow for AI that is fully people-centered and responsible. This approach would also support the International Labor Organisation's (ILO) Decent Work agenda that helps advance all employees' working conditions in varied working environments.

## Data availability statement

The raw data supporting the conclusions of this article will be made available by the authors, without undue reservation.

## Ethics statement

The studies involving humans were approved by University of Surrey (UK) Ethics Committee. The studies were conducted in accordance with the local legislation and institutional requirements. The participants provided their written informed consent to participate in this study.

## Author contributions

A-SW: Data curation, Formal analysis, Writing – original draft. SE: Conceptualization, Methodology, Writing – review & editing. ED: Conceptualization, Methodology, Resources, Writing – review & editing.
